# No time for drifting: Comparing performance and applicability of signal detrending algorithms for real-time fMRI

**DOI:** 10.1016/j.neuroimage.2019.02.058

**Published:** 2019-05-01

**Authors:** R. Kopel, R. Sladky, P. Laub, Y. Koush, F. Robineau, C. Hutton, N. Weiskopf, P. Vuilleumier, D. Van De Ville, F. Scharnowski

**Affiliations:** a Department of Radiology and Medical Informatics, CIBM, University of Geneva, Geneva, Switzerland; b Institute of Bioengineering, Ecole Polytechnique Fédérale de Lausanne (EPFL), Lausanne, Switzerland; c Department of Psychiatric, Psychotherapy and Psychosomatics, Psychiatric Hospital, University of Zürich, Zürich, Switzerland; d Social, Cognitive and Affective Neuroscience Unit, Department of Basic Psychological Research and Research Methods, Faculty of Psychology, University of Vienna, Vienna, Austria; e Department of Radiology and Medical Imaging, Yale University, New Haven, USA; f Laboratory for Behavioral Neurology and Imaging of Cognition, Department of Neuroscience, University Medical Center, Geneva, Switzerland; g Geneva Neuroscience Center, Geneva, Switzerland; h Wellcome Trust Centre for Neuroimaging, UCL Institute of Neurology, University College London, London, UK; i Department of Neurophysics, Max Planck Institute for Human Cognitive and Brain Sciences, Leipzig, Germany; j Neuroscience Center Zürich, University of Zürich and Swiss Federal Institute of Technology, Winterthurerstr. 190, 8057, Zürich, Switzerland; k Zürich Center for Integrative Human Physiology (ZIHP), University of Zürich, Winterthurerstr. 190, 8057, Zürich, Switzerland

**Keywords:** Real-time fMRI, Detrending, Temporal stability, Signal drifts, Incremental GLM, Moving average

## Abstract

As a consequence of recent technological advances in the field of functional magnetic resonance imaging (fMRI), results can now be made available in real-time. This allows for novel applications such as online quality assurance of the acquisition, intra-operative fMRI, brain-computer-interfaces, and neurofeedback. To that aim, signal processing algorithms for real-time fMRI must reliably correct signal contaminations due to physiological noise, head motion, and scanner drift. The aim of this study was to compare performance of the commonly used online detrending algorithms exponential moving average (EMA), incremental general linear model (iGLM) and sliding window iGLM (iGLM^window^). For comparison, we also included offline detrending algorithms (i.e., MATLAB's and SPM8's native detrending functions). Additionally, we optimized the EMA control parameter, by assessing the algorithm's performance on a simulated data set with an exhaustive set of realistic experimental design parameters. First, we optimized the free parameters of the online and offline detrending algorithms. Next, using simulated data, we systematically compared the performance of the algorithms with respect to varying levels of Gaussian and colored noise, linear and non-linear drifts, spikes, and step function artifacts. Additionally, using *in vivo* data from an actual rt-fMRI experiment, we validated our results in a *post hoc* offline comparison of the different detrending algorithms. Quantitative measures show that all algorithms perform well, even though they are differently affected by the different artifact types. The iGLM approach outperforms the other online algorithms and achieves online detrending performance that is as good as that of offline procedures. These results may guide developers and users of real-time fMRI analyses tools to best account for the problem of signal drifts in real-time fMRI.

## Highlights

• fMRI time series are almost always affected by signal drifts.
• Signal drifts can be reduced in real-time using different correction methods.
• Robustness of these methods was tested in the presence of different artifacts.
• Incremental GLM (iGLM, iGLM^window^) were found optimal in most cases.

## 1 Introduction

Recent technological advances in the field of functional magnetic resonance imaging (fMRI) have made it possible to obtain the information about brain activations in real-time. This allows for applications such as online quality assurance of the acquisition, intra-operative fMRI (Gasser et al., 2005; Hirsch et al., 2000; Kuhnt et al., 2012; Matthews et al., 2006; Nimsky, 2011a, 2011b), brain-computer-interfaces (Andersson et al., 2011, 2013; Eklund et al., 2009; Naci et al., 2012; Sorger et al., 2009, 2012; Yoo et al., 2004), and neurofeedback (deCharms, 2007, 2008; Sitaram et al., 2017; Sulzer et al., 2013; Weiskopf, 2012; Weiskopf et al., 2004b, 2007). For real-time fMRI, data acquisition, preprocessing, and analysis need to be optimized for speed. General improvements in the field of fMRI, such as specific imaging sequences that allow to acquire high quality data within a very short time (Posse et al., 1999, 2012; Speck and Hennig, 1998; Weiskopf et al., 2004a, 2005) and the availability of higher magnetic field strengths have dramatically increased sensitivity of present-day fMRI (Duyn, 2012; Hahn et al., 2013; Sladky et al., 2013, 2018; van der Zwaag et al., 2009; Yacoub et al., 2008) and real-time fMRI methods (Baecke et al., 2015; Grone et al., 2015; Koush et al., 2011, 2013, 2014). In addition, real-time fMRI data analysis benefits from steadily increasing computational power (Moore, 1965), from the optimization of real-time analysis algorithms (Hinds et al., 2011; Koush et al., 2012; Magland et al., 2011), and from the adaptation of sophisticated data analysis techniques for real-time purposes (Esposito et al., 2003; Hollmann et al., 2011; Koush et al., 2013; LaConte et al., 2007; Sitaram et al., 2011; Zilverstand et al., 2014).

**Fig. 1:**
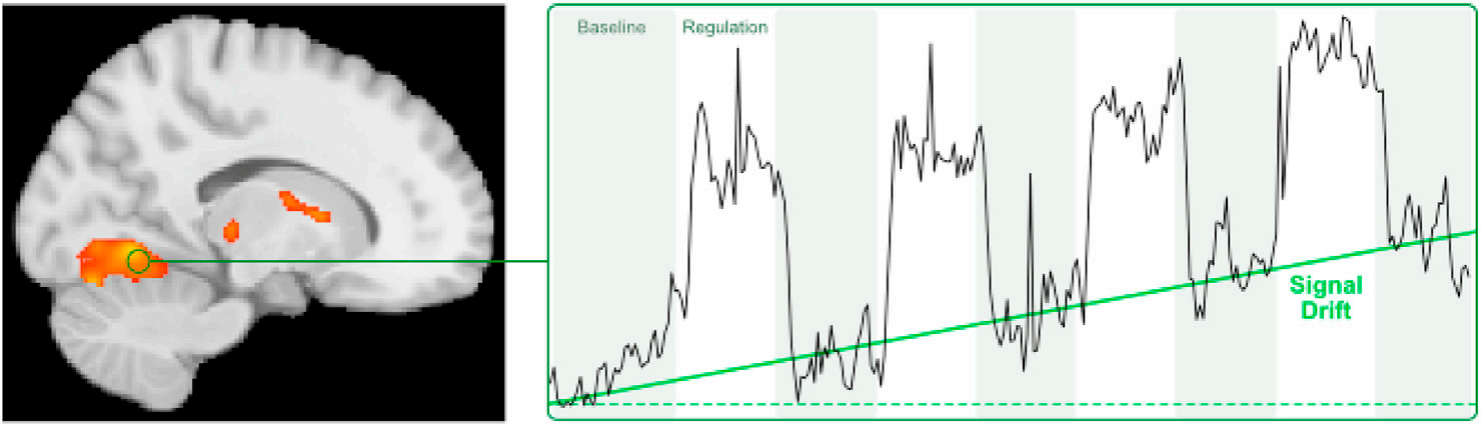
**Linear signal drift.** Scanner instabilities or head motion artifacts can cause signal drifts that compromise data quality in fMRI. In conventional fMRI experiments, such drifts can be corrected during the offline analysis, but for real-time applications, such drifts need to be detected and corrected during scanning.

Despite these constant advances in the field of real-time fMRI, unresolved challenges limit its robustness and its applicability. These challenges include magnetic field inhomogeneities, physiological noise, head motion artifacts, and scanner drifts. These problems can severely compromise data quality in fMRI in general, and they pose specific constraints on real-time fMRI. For example, it is generally advisable in fMRI to avoid areas that exhibit strong magnetic susceptibility artifacts because such artifacts limit the contrast-to-noise ratio. However, in conventional fMRI this can partially be compensated for by averaging over more acquisitions or increasing spatial resolution at the cost of temporal resolution (Morawetz et al., 2008; Robinson et al., 2004), which is not suitable for real-time fMRI methods that require high temporal accuracy. Likewise, in conventional fMRI physiological parameters like breathing or heart rate can be recorded and can then be taken into account during the offline data analysis. Another concern is the presence of physiological noise artifacts (Misaki et al., 2015), i.e., respiration and cardiac signal, and head motion as the single largest source of noise in fMRI. Considerable efforts have been made to correct for these artifacts in real-time (Koush et al., 2012, 2017a; Lee et al., 1996; Scheinost et al., 2013; Thesen et al., 2000). Despite all these improvements of the last years it is a well-known problem that residual slow head movements and scanner instabilities (e.g., due to gradual changes in temperature of the components and strength of the local magnetic field) can result in low frequency signal drifts that debilitate fMRI data analysis (Fig. 1).

Previously, several approaches have been proposed to correct for these signal drifts in real-time.

The aim of the present study is to quantify the negative effects of linear and non-linear signal drifts and investigate the performance of different detrending approaches. Most important for univariate BOLD-based rt-fMRI, are the incremental general linear model (iGLM) (Bagarinao et al., 2003; Nakai et al., 2006) and the exponential moving average (EMA) algorithms (Cui et al., 2010; Koush et al., 2012; Roberts, 1959), which were compared in this study. For multi-variate pattern analysis based rt-fMRI, however, these voxel-wise detrending approaches might be sub-optimal (Lee et al., 2015). Correlation-based approaches, such as many functional connectivity methods, could benefit from directly implementing detrending during sliding-window correlation analysis (Gembris et al., 2000). Additionally, some metrices derived from resting state fMRI connectivity (i.e., ALFF, fALFF, and hfALFF) are affected differently by signal drifts and need to be processed accordingly (Woletz et al., 2018).

## 2 Methods

### 2.1 Detrending algorithms applicable for real-time fMRI

In contrast to conventional fMRI data analysis, a fundamental challenge of rt-fMRI data analysis is that estimations have to be based on incomplete data. One common approach in rt-fMRI is to restrict analysis to the most recent acquisitions, i.e., the sliding window approach. In this case, potential effects of signal drifts are smaller for data acquired closer in time, the piecewise analysis of the sliding window approach reduces the problem of signal drifts.

**Fig. 2:**
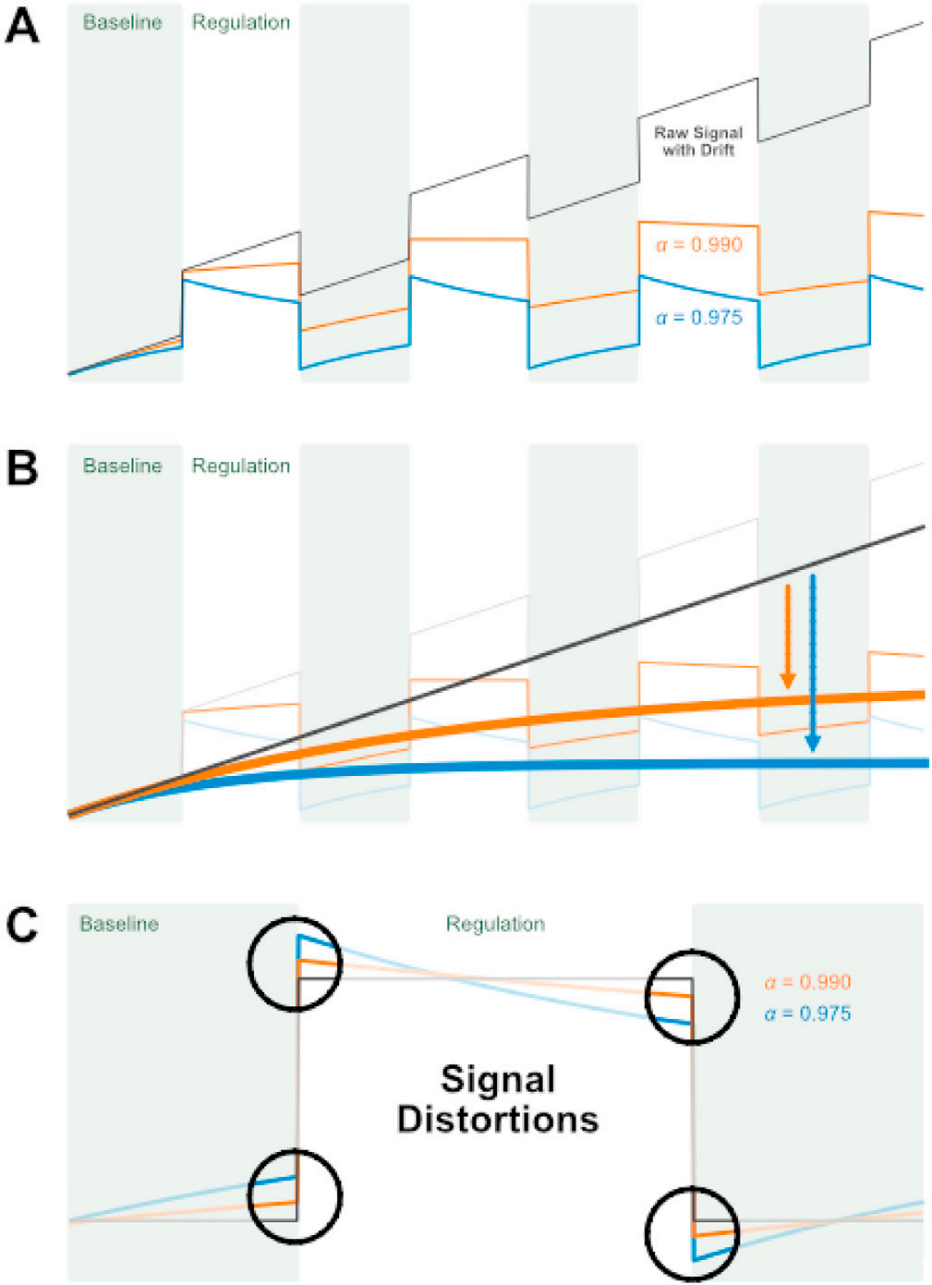
**Methodological challenges for the EMA method.** Shown is the functioning of the EMA algorithm for simulated data that corresponds to an fMRI block design plus added linear drift (black line). **A.** The performance of the EMA is determined by the free parameter *α*. An *α* close to 1 causes slow conversion with little signal distortions (*α* = 0.990, orange line), and a smaller *α* causes fast conversion but also larger signal distortions (*α* = 0.975, blue line). **B** shows how the choice of *α* affects the convergence interval. **C** shows how the choice of *α* affects distortions of the actual signal.

Alternatively, detrending algorithms can be used to correct the signal drift in real-time. For example, one could estimate the feedback signal by computing an incremental general linear model (iGLM) fit to the fMRI time series (Bagarinao et al., 2003; Nakai et al., 2006), which allows for flexibly removing unwanted signals including drifts. Others used real-time adaptations of the most commonly used algorithm to correct linear drifts, the exponential moving average (EMA) algorithm (Cui et al., 2010; Koush et al., 2012; Roberts, 1959). The EMA method corresponds to an online high-pass-filtering, that can be easily implemented, and that is computationally sufficiently fast for real-time applications (Fig. 2). The efficiency of the EMA algorithm is determined mainly by the free parameter α, which controls the steepness of the filter. Magland et al. (2011) compared the EMA performance to two retrospective real-time detrending methods: (1) simple cycle mean subtraction where the mean of each 40-frame cycle was subtracted from the data before the analysis, (2) linear detrending where sliding-window linear fits were subtracted from the data. They found that the EMA algorithm is suboptimal, mostly because the free parameter *α* has to be chosen a priori although it strongly depends on the signal characteristics which in a real-time setting are only partially known. An *α* close to 1 causes the EMA algorithm to converge slowly to steeper trends, which bears the disadvantage that the trend is initially not corrected for. On the other hand, such an α level avoids distortions of the actual signal (Fig. 2). A smaller *α* causes the EMA algorithm to converge faster, thus ensuring that the data is detrended right from the beginning of the experiment (Fig. 2). On the other hand, such *α* levels might distort the actual signal (Fig. 2; green line). Both, the convergence interval and the actual signal distortions can affect the reliability of the real-time fMRI signal.

This example shows that the choice of free parameters in the detrending algorithms does affect their performance. Unfortunately, methods for carefully selecting them, or an explanation for the choice of reported parameters is rarely provided. Likewise, a comprehensive performance comparison between different detrending approaches that are suitable for real-time fMRI has not been done so far. Here, we used synthetic and real data containing different noise sources and signal distortions and real data to compare performance of state-of-the-art real-time detrending approaches, i.e., the EMA, iGLM and iGLM^window^ algorithms. Before comparing their performance, we first optimized their free parameters for the given test data. For the EMA, this optimization included finding the most suitable *α* parameter based on the known signal characteristics related to the study design. The goal of the optimization of the EMA was to remove signal drifts early on (i.e., fast conversion), without distorting the actual signal. We then compared performance of the online algorithms to well-established retrospective detrending methods, as implemented in MATLAB and SPM8. It should be noted that MATLAB's detrend is limited to a (piece-wise) linear detrending by subtracting from the data the result of a least-squares fit of a straight line to the data. Thus, this approach is very robust and easy to implement but not designed to correct for sudden (artifactual) signal changes. In contrast, the other detrending methods tested in this study allow for a more flexible high-pass filtering. Quantitative quality measures demonstrate robust removal of artifacts, improved signal quality for real-time fMRI purposes, and allow for clear recommendations as to what method works best and should be used.

### 2.2 Optimizing free parameters of the detrending algorithms

Selection of the most suitable parameters of each detrending algorithm was done with respect to the block design used here (see below). Specifically, we chose the parameters that showed the highest correlation between the original synthetic data and the output signals of the detrending algorithms.

Below is a list of the free parameters for each algorithm, and the range of values we have evaluated for finding the optimal parameter setting:

1. *EMA; α* values varying between 0.97 and 0.995 in steps of 0.005.
2. *Incremental GLM* with a design matrix comprising (1) a constant term, (2) a boxcar function according to the task block design, (3) a linear drift term, and (4) a set of seven high-pass filter terms (Bagarinao et al., 2003); no free parameter.
3. *Sliding window iGLM* (iGLM^window^) (Nakai et al., 2006; window length n = 50 samples); sliding window lengths between n = 10 to 60 samples in steps of 5.
4. *MATLAB's detrend.m* (MATLAB); computes a linear regression of the entire or pieces of the time course, no free parameter.
5. *SPM8's spm_dctmt.m* (SPM8); corresponds to a high-pass filter based on a set of discrete cosine transform (DCT) functions; the free parameter is the cutoff period that determines the order of the SPM8 Detrend, whose values we evaluated for n = 3, 4, 5, 6, and 7.

### 2.3 Comparison of the detrending algorithms using simulated data

The detrending algorithms were compared using standard quality measures applied to synthetic data with a large range of commonly found sources of noise. Synthetic fMRI data was generated in MATLAB 8.3.0 (Mathworks Inc., Natick, MA, USA) by convolving boxcar functions with SPM8's canonical hemodynamic response function (HRF) (Wellcome Trust Centre for Neuroimaging, Queen Square, London, UK; http://www.fil.ion.ucl.ac.uk/). The structure of the block design had three variable parameters: number of samples in each upregulation block (*n*_REG_), number of samples in each baseline block (*n*_*BL*_), and number of block repetitions (*n*_*BLOCKS*_). Using this general form of an fMRI time course, we generated a set of 36 different synthetic fMRI data, where *n*_*RE*_ and *n*_*BL*_ were independently varied from 10 to 60 time points in steps of 10 and *n*_*BLOCKS*_ = 15.

**Fig. 3:**
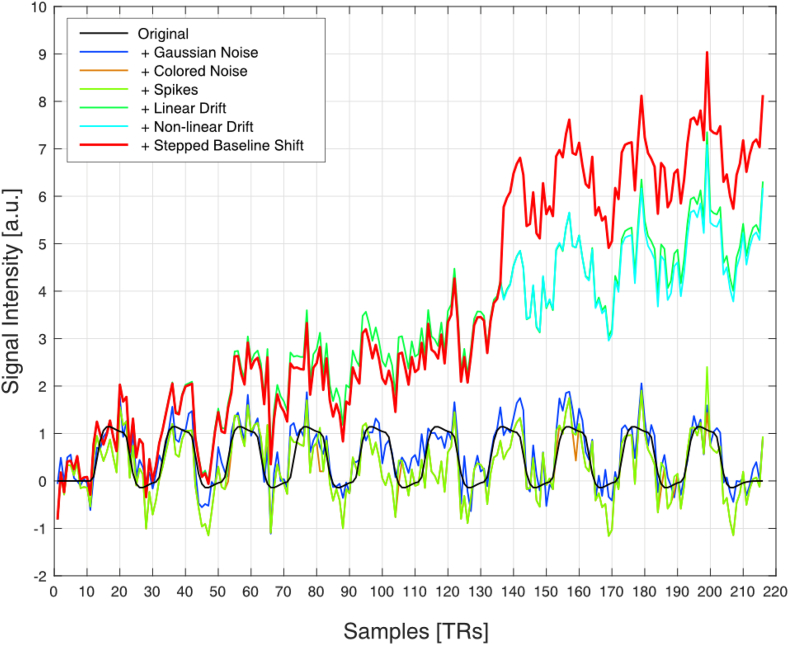
Representation of one typical synthetic time course. Starting with a simulated BOLD response (black), several noise sources (Gaussian, blue; colored, orange; spikes, light green) and temporal instabilities (linear trend, green; non-linear trend, cyan; stepped baseline shift, red) were sequentially added. The red time course thus represents a time course that is affected by artifacts commonly found in (real-time) fMRI data.

To test the effects of different sources of noise that are commonly found in (real-time) fMRI experiments, the synthetic fMRI data was degraded with each of the following six different types of noise (Fig. 3):

1. Gaussian random noise, where the signal-to-noise ratio (SNR) was varied from 5 dB to 25 dB in steps of 5 dB. For each SNR level, 20 synthetic noise signals were generated.
2. Linear drifts with a constant slope varying from 0.05 to 0.3 in steps of 0.05.
3. Non-linear drifts generated by using existing resting state fMRI data of a single participant filtered by a low pass filter with a cutoff frequency at 128 Hz.
4. Colored noise obtained from resting state fMRI data of a single participant.
5. Spikes composed of 3 delta functions of random magnitude that were added randomly to the synthetic data. 20 synthetic noise signals were generated.
6. Step function with a magnitude of 5 that was added at random time points to the synthetic time series. 20 synthetic noise signals were generated.

To compare the performance of the detrending algorithms, we evaluated performances of all methods using the synthetic real-time fMRI data described above. We evaluated two commonly used block lengths, one with BL and REG equal to 10 samples, and one where they both consist of 40 samples. As performance measure, we used the correlation coefficient between the original time-course without noise, and the detrended time-course (i.e., the output signal of the de-trending algorithms). Each of the detrending algorithms was operated using the optimal parameters based on the conclusions from the previous section.

### 2.4 Comparison of the detrending algorithms using *in vivo* real-time fMRI data

In addition, to validate the results from the simulated data, we applied the detrending algorithms on *in vivo* data from an openly available online repository (Koush et al., 2017b). We used the NF_PSC_int data, where a participant performed a neurofeedback run to learn control over two VOIs within the primary visual cortex. The neurofeedback run consisted of seven 20 s regulation blocks followed by 4 s of neurofeedback display (i.e., intermittent feedback) and 16 s baseline blocks, resulting in a total duration of 20 s + (20 s + 4 s + 16 s)*7 = 300 s. The experiment was performed at the Brain and Behavior Laboratory (University of Geneva) on a 3T MR scanner (Trio Tim, Siemens Medical Solutions, Germany). Functional images were acquired with a single-shot gradient-echo T2*-weighted EPI sequence with 150 scans (32 channel receive head coil, TR = 1970 ms, volume size = 74 × 74 × 36 voxels, isotropic 3 mm voxel, flip angle *α* = 75°, bw = 1572 Hz/pixel, TE = 30 ms). The first five EPI volumes were discarded to account for T1 saturation effects. The raw, unprocessed time series of the left and right VOIs were used to test the efficacy of the detrending algorithms. To quantify performance of the online detrending algorithms, we calculated the parameter estimates (*ß*) and *t*-values from a general linear model estimation with MATLAB's *fitlm* function using the experimental paradigm and constant term as design matrix.

Runtime was measured using MATLAB's timing functions (*tic, toc*) for time series covering the first 10 and all 150 TRs and then averaged for both VOIs. Calculations were performed on a 2017 MacBook Pro, 3.1 GHz Intel Core i5, 16 GB LPDDR3 2133 MHz RAM computer using MATLAB 2017b. Measurements were repeated 9 times in randomized order and revealed no relevant changes in runtime.

## 3 Results

### 3.1 Optimization of detrending algorithms

**Fig. 4:**
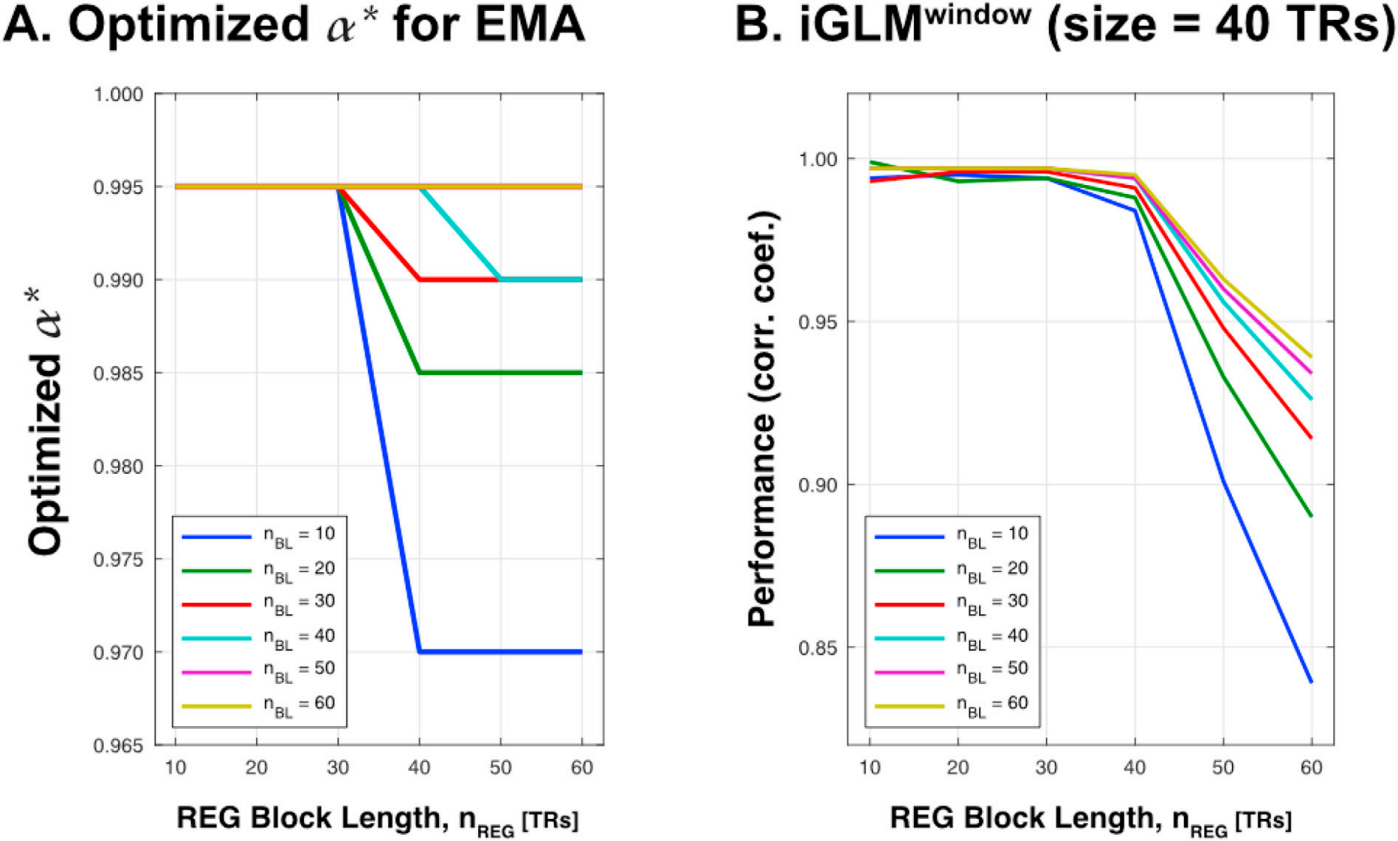
**Optimization of free parameters of EMA and iGLM**^window^**. A.** For shorter blocks, the EMA performs best for *α* = 0.995. With longer REG blocks, a smaller *α* performs better. The commonly used *α* = 0.975 is suboptimal in all cases. **B.** An iGLM^window^ with a given window length of 40 samples performs well as long as the block length of the REG does not exceed the window size.

Before comparing performance of the different detrending approaches, we optimized their free parameters for the experimental design used here (i.e., *n*_*BL*_ and *n*_*REG*_ block lengths of 10 and 40 TRs). The optimal free parameters are reported in Table 1. We also illustrate the dependency of the optimal free parameters on *n*_*REG*_ and *n*_*BL*_ block lengths (Fig. 4). For the EMA, an *α* of 0.995 performs well, unless the design contains longer *REG* blocks and short *BL* blocks, as it is often the case for neurofeedback experiments. For such designs, a smaller *α* should be used. For the iGLM^window^, it is crucial that the size of the sliding window exceeds that of the block lengths. Otherwise, a noticeable artifact mainly during the transition between *BL* and *REG* degrades detrending performance (Fig. 4). For SPM8's *spm_dctmtx.m* function, the free parameter cutoff period did not considerably affect detrending performance.

**Table 1:** Optimized free parameters of detrending algorithms EMA, iGLM^window^. Note: Standard iGLM and MATLAB's detrend do not possess free parameters and parameter choice in SPM8's *spm_dctmtx.m* function had no relevant consequence for the detrending performance.

	REG [TRs]	BL [TRs]	Parameters
EMA	10	10	*α* = 0.995
	40	40	*α* = 0.995
iGLM^window^	10	10	window length = 30 TRs
	40	40	window length = 60 TRs

EMA – exponential moving average; iGLM^window^ – sliding window incremental GLM.

### 3.2 Comparison of detrending performance

Detrending performance was estimated by correlating the original unbiased source signal with the signal after detrending. Assessing performance of all six algorithms in the presence of various levels of noise and drift components, our results show that the iGLM^window^ is the most robust online algorithm to eliminate drift components and artifacts without distorting the signal itself. It has almost similar performance benchmarks as retrospective offline algorithms. Performance analysis was conducted based on the optimal parameters of each algorithm and typical real-time fMRI noise sources.

**Fig. 5:**
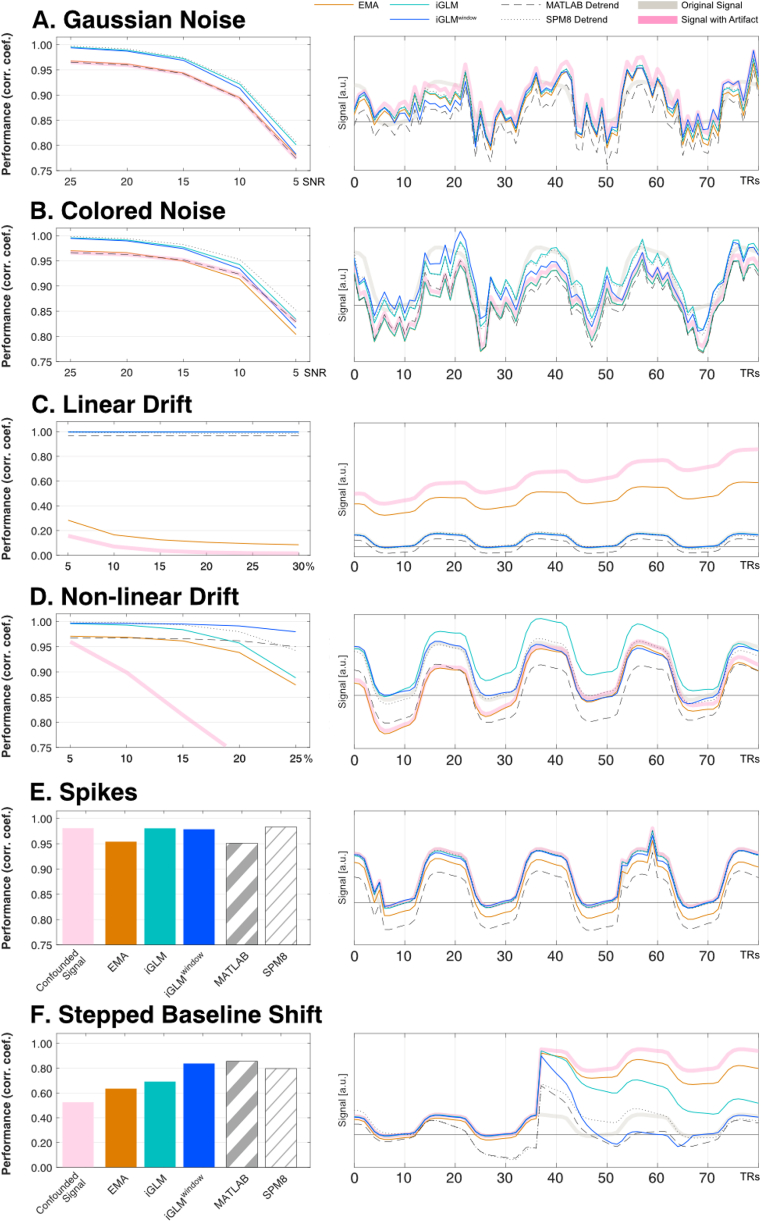
**Comparison of detrending performance** average over all simulations for n_BL_ = n_REG_ = 10 and 40 TRs and exemplary time courses from the extensive simulation dataset, n_BL_ = n_REG_ = 10 TRs) for Gaussian noise (A), colored noise (B), linear drift (C), non-linear drift (D), spikes (E), and stepped baseline shifts (F).

To better investigate the individual characteristics of each method, we have tested the impact of each noise type separately. Our findings suggest that the different noise sources affect the examined algorithms differently. For white Gaussian and colored noise, the best real-time online algorithm was iGLM, yet the other algorithms were only affected by these noise sources to a minor degree (Fig. 5A and B). As expected, offline MATLAB detrending did not alter the confounded signal, i.e., it did not affect the high-frequency signal components introduced by the additive Gaussian and colored noise. In this scenario, the GLM-based methods including SPM8's detrending, showed to reduce the amount of high-frequency noise, indicated by the higher correlation with the unbiased source signal, due to a small amount of temporal smoothing of the time series.

In contrast, for low-frequency linear drifts, performance of the EMA degraded as the slope of the drift increased, whereas iGLM and iGLM^window^ performed much better and accounted equally well for various levels of linear drift interference (Fig. 5C). Interestingly, the EMA performance was also affected by the size of the block design with better performance when the block size increased.

Non-linear drifts are an additional type interference that can be found in fMRI signals. We found that the iGLM^window^ outperformed all other online and offline algorithms in removing this noise source (Fig. 5D). This high performance is due to the ability of the iGLM^window^ to quickly adapt to changes. However, due to the nature of this algorithm, it does not carry knowledge about the previous noise characteristics outside the time window used for estimation. Overall, all algorithms handle non-linear drifts well, and major performance differences occur only for high levels of drift.

For spikes, we only observed negligible performance differences between all tested algorithms (Fig. 5E). This was expected, because detrending methods aim at removing other signal artifacts than spikes. In fact, correlation analyses showed that iGLM^(window)^ and SPM8's detrend were almost identical to the confounded signal.

Stepped baseline shifts are a more fundamental problem for detrending and should be corrected using, e.g., real-time realignment to reduce the effects of head motion, the most likely source of these artifacts. In our simulations, stepped baseline shifts introduced a linear drift in EMA-corrected data that persisted for the whole time series for step sizes >200% of the simulated BOLD response. In iGLM-corrected data, on the other hand, a negative yet stable baseline shift was observed. Both biases can lead to an underestimation of the actual BOLD response in traditional GLM fMRI analyses. For these artifacts, iGLM^window^ provided the most reliable compensation. Using the suggested window length, we observed a stabilization of the signal before the next task block. Naturally, for increasing window length, the iGLM^window^ results approached the iGLM results.

Overall, the iGLM^window^ works better than the other online detrending approaches and performs as well as the offline detrending approaches that are implemented in Matlab and SPM.

An exemplary time course to visually illustrate how the different algorithms correct for the respective signal artifacts is shown in Fig. 5.

### 3.3 Comparison of the detrending algorithms using *in vivo* real-time fMRI data

**Fig. 6:**
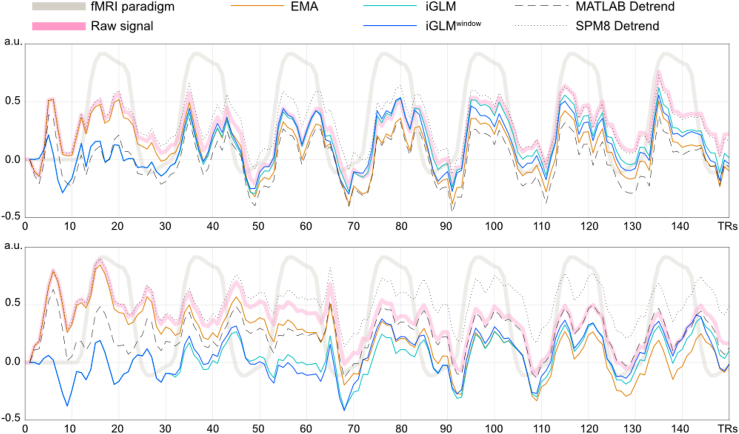
Illustration of detrending performance of *in vivo* real-time data from left VOI (first row) and right VOI (second row).

In both time series we observed the best detrending performance by the iGLM/iGLM^window^ algorithms (Fig. 6). For the first time series (left VOI), parameter estimates and *t*-values for all algorithms were comparable to SPM8's detrending, with iGLM^window^ and iGLM performing better than EMA. For the second time series, parameter estimates were considerably lower for the EMA, while iGLM and iGLM^window^ performance matched or exceeded SPM8's detrending (Table 2). The same (optimal) parameters were used as in Table 1 and Fig. 5.

**Table 2:** **Comparison of detrending performance of in vivo real-time data**. In general, and for the right VOI in particular, we observed a better performance of iGLM/iGLM^window^ compared to the EMA method. Parameter estimates (*ß*) and *t*-values resulted from GLM estimation (MATLAB's *fitlm* using the experimental paradigm and constant term as design matrix). Average runtime was measured for the first 10 and all 150 TRs of a single time series and a volume with 90 × 90 × 15 voxels.

Method	left VOI	right VOI	Timeseries n = 10, 150 TRs	Volume n = 10, 150 TRs
EMA	*ß* = +1.58, *t* = 10.64	*ß* = +0.51, *t* = 2.97	<1 ms, <1 ms	289 ms, 760 ms
iGLM	*ß* = +1.68, *t* = 11.88	*ß* = +1.27, *t* = 5.43	3 ms, 4 ms	456 ms, 890 ms
iGLM^window^	*ß* = +1.85, *t* = 13.73	*ß* = +1.32, *t* = 6.29	1 ms, 1 ms	482 ms, 508 ms
MATLAB Detrend	*ß* = +1.78, *t* = 11.12	*ß* = +1.21, t = 4.80	<1 ms, <1 ms	>1000 ms
SPM8 Detrend	*ß* = +1.83, *t* = 12.86	*ß* = +1.23, t = 5.32	<1 ms, <1 ms	472 ms, 884 ms

Another central aspect for real-time applications is computation speed of the individual algorithms. For the whole time series, due to its simplicity, the EMA algorithm was the fastest of all tested online detrending algorithms (t < 1 ms), compared to iGLM (t < 4 ms) and iGLM^window^ (t < 2 ms). In comparison, MATLAB detrending of the time series took t < 1 ms (Table 2). Runtime was determined by MATLAB's internal clock functions (*tic*, *toc*) during detrending of the *in vivo* rt-fMRI time series.

## 4 Discussion

Signal drifts pose serious challenges for real-time fMRI data analyses. Here we used an extensive data set of simulated fMRI time series that were contaminated with realistic artifacts that are found in real-time fMRI setups (i.e., linear and non-linear drift, Gaussian and non-Gaussian noise, spikes and stepped baseline shifts). We simulated several different fMRI paradigms with baseline and regulation block lengths that are commonly used in real-time fMRI and compared the performance of the most commonly used online and offline detrending algorithms.

Out of all the algorithms tested, the GLM-based filtering approach performed best. The windowed iGLM algorithm, in particular, provided optimal online detrending that performed similar to retrospective offline methods in almost all tested cases. Furthermore, the iGLM provides additional flexibility and advantages as other known nuisance regressors such as subject movement, physiological noise, and white matter as well as CSF time courses can easily be included into the model. This might be advisable in particular for ultra-high field applications (Andersson et al., 2011, 2012). However, a disadvantage of the iGLM-based method is that it is more complex to implement than, for example, the EMA algorithm. On the other hand, the iGLM is already implemented in freely available real-time fMRI analysis toolboxes, such as OpenNFT (Koush et al., 2017a) and BART (Hellrung et al., 2015), and MATLAB implementations of the respective algorithms are shared in the supplement of this publication.

When using the EMA in real-time fMRI, design specific considerations should be addressed to ensure performance detrending. Especially for designs with longer task/regulation blocks and short baseline blocks, which are often used in neurofeedback experiments, the commonly used smoothing level of α = 0.975 is suboptimal, and larger α levels are recommended (Fig. 4). In cases of very long task/regulation blocks (i.e. n_REG_>40 TRs) and very short baseline blocks (i.e. n_BL_ = 10 TRs), α levels of 0.970 would be advisable. Even though iGLM/iGLM_window_ algorithms have shown to be the most reliable, the standard EMA algorithm with an adapted control parameter α can be a suitable alternative due to its simplicity and fast computability, in particular if no GLM results are required for neurofeedback.

These results were obtained using simulated fMRI time series and were validated with empirical fMRI data. Actual fMRI data is compromised by several unknown noise sources and does not allow for establishing a ground truth about the neuronal signal dynamics responsible for the BOLD response. In contrast, simulated data allows for a detailed evaluation of the individual detrending approaches with respect to the different noise sources. Despite their ability to reduce low-frequency drifts considerably, detrending algorithms are obviously not a *panacea* for all possible signal artifacts. Particularly high-frequency noise and signal spikes are not mitigated by applying detrending algorithms. Such artifacts can be reduced by the application of, e.g., a low-pass Kalman filters (Koush et al., 2012). Likewise, the impact of stepped baseline shifts due to subject movement can be reduced by prospective motion correction (Maclaren et al., 2013) and online real-time realignment (Koush et al., 2017a).

A limitation of this study is that we only investigated block designs. The reason this work focuses on block designs is that almost all real-time fMRI studies use block designs, and the signal distortions discussed here pose fewer problems for event related designs. Additionally, the simulated and *in vivo* data have been detrended *post hoc*, i.e., not during an ongoing rt-fMRI experiment. However, the online detrending algorithms are usable in real world rt-fMRI settings because they provide a sequentially updated, detrended time series (Hellrung et al., 2015; Hinds et al., 2011; Koush et al., 2012, 2017a; Magland et al., 2011). Also, the way we evaluated the algorithms corresponds to how the data would be processed in an rt-fMRI experiment, i.e., sequentially.

In conclusion, our results show that all detrending algorithms perform well, even though they are differently affected by the different artifact types. For optimal performance up to the level of offline detrending algorithms the (windowed) iGLM approach should be used.
